# Growth of Wafer‐Scale Single‐Crystal 2D Semiconducting Transition Metal Dichalcogenide Monolayers

**DOI:** 10.1002/advs.202307839

**Published:** 2024-01-02

**Authors:** Jitendra Singh, Nadiya Ayu Astarini, Meng‐Lin Tsai, Manikandan Venkatesan, Chi‐Ching Kuo, Chan‐Shan Yang, Hung‐Wei Yen

**Affiliations:** ^1^ Department of Materials Science and Engineering National Taiwan University of Science and Technology Taipei City 106335 Taiwan; ^2^ Department of Physics Udit Narayan Post Graduate College Padrauna Kushinagar Uttar Pradesh 274304 India; ^3^ Department of Molecular Science and Engineering Institute of Organic and Polymeric Materials National Taipei University of Technology Taipei City 106344 Taiwan; ^4^ Institute and Undergraduate Program of Electro‐Optical Engineering National Taiwan Normal University Taipei City 11677 Taiwan; ^5^ Department of Materials Science and Engineering National Taiwan University Taipei City 106319 Taiwan

**Keywords:** 2D materials, chemical vapor deposition, field effect transistors, single crystal, transition metal dichalcogenides

## Abstract

Due to extraordinary electronic and optoelectronic properties, large‐scale single‐crystal two‐dimensional (2D) semiconducting transition metal dichalcogenide (TMD) monolayers have gained significant interest in the development of profit‐making cutting‐edge nano and atomic‐scale devices. To explore the remarkable properties of single‐crystal 2D monolayers, many strategies are proposed to achieve ultra‐thin functional devices. Despite substantial attempts, the controllable growth of high‐quality single‐crystal 2D monolayer still needs to be improved. The quality of the 2D monolayer strongly depends on the underlying substrates primarily responsible for the formation of grain boundaries during the growth process. To restrain the grain boundaries, the epitaxial growth process plays a crucial role and becomes ideal if an appropriate single crystal substrate is selected. Therefore, this perspective focuses on the latest advances in the growth of large‐scale single‐crystal 2D TMD monolayers in the light of enhancing their industrial applicability. In the end, recent progress and challenges of 2D TMD materials for various potential applications are highlighted.

## Graphical abstract

This perspective focuses the latest advances on the growth of large‐scale single‐crystal two‐dimensional (2D) transistion metal dichalcogenide (TMD) monolayers in the light of enhancing their industrial applicability. Recent progress and challenges of 2D TMD materials for various potential applications are highlighted.

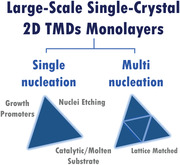

## 1 Introduction

The growth of large‐scale ultrathin two‐dimensional (2D) monolayers of semiconducting transition metal dichalcogenides (TMDs) has emerged as an important topic and generated significant interest in fundamental research and industrial applications. They could help produce cutting‐edge electronic and optoelectronic devices, providing good flexibility, excellent bandgap tunability, high mobility, and considerable thermal stability.^[^ ^1^, ^2^, ^3^, ^4^, ^5^, ^6^, ^7^, ^8^, ^9^, ^10^ ^]^ However, the controllable growth of high‐quality single‐crystal 2D monolayers still needs to be applied in potential applications.^[^ ^11^, ^12^ ^]^ The performance of TMD‐based devices might be substantially influenced by the grain boundaries and crystal defects, which commonly act as scattering/trapping centers for charge carriers and hinder device carrier mobility.^[^ ^13^ ^]^ To overcome these bottlenecks, research has been conducted on directly synthesizing highly oriented large‐scale/domain‐size TMD single crystals with low deformity on *c*‐plane sapphire substrates.^[^ ^14^, ^15^, ^16^ ^]^ In 2020, highly oriented epitaxy of wafer‐scale monolayers of MoS_2_ on sapphire has first been demonstrated on 4‐inch wafers to exhibit carrier mobility of ≈70 cm^2^ V·s^−1^ and on/off ratio of ≈10^9^.^[^ ^15^ ^]^ Recently, 4‐inch flexible wafer scale monolayer MoS_2_ field‐effect transistors (FETs) have been demonstrated by employing polyethylene terephthalate (PET) substrates and atomic layer deposition (ALD) deposited HfO_2_ as dielectric layers, showing average mobility of ≈70 cm^2^ V·s^−1^, on/off ratio of 5 × 10^7^, and subthreshold swing of 83 mV·dec^−1^ for over 500 randomly picked devices.^[^ ^16^ ^]^ Bulk crystalline TMD, such as MoS_2_ and WS_2_, can be obtained from molybdenite and tungstenite minerals. Due to the weak van der Waals coupling between layers, monolayer or few‐layer TMD can be prepared through the exfoliation process using scotch tape or liquid‐phase exfoliation.^[^ ^17^, ^18^ ^]^ In bulk, most TMD semiconducting materials reveal indirect bandgap, whereas monolayers exhibit direct bandgap due to the quantum confinement effect.^[^ ^19^ ^]^ This unique layer‐dependent characteristic of 2D TMDs opens a new window for various possible applications.^[^ ^20^ ^]^ For example, it is reported that the bandgap of 2D TMDs can be varied effortlessly through strain engineering.^[^ ^21^, ^22^ ^]^ The enormously increased Coulomb interaction in the direct bandgap TMD monolayer makes it an ideal model for many‐body‐associated quasiparticles’ fundamental properties in condensed matter physics.^[^ ^23^ ^]^ Such particular functionalities of TMD monolayers make them appropriate candidates for designing various electronic and optoelectronic devices, including but not limited to field‐effect transistors, photodetectors, light‐emitting diodes, excitonic circuits, and viable photo‐catalysts.^[^ ^24^, ^25^, ^26^, ^27^ ^]^

Top‐down exfoliation techniques of TMDs result in microscale flakes along with an arbitrary dispersion of film thickness, which can only be applicable in fundamental research.^[^ ^28^, ^29^ ^]^ Over the years, various bottom‐up strategies have been devoted to producing large‐scale TMD films and monolayers. These strategies include molybdenum oxide sulfurization, pulsed layer deposition (PLD), and chemical vapor deposition (CVD).^[^ ^30^, ^31^, ^32^ ^]^ As‐grown TMD films using the above techniques exhibit polycrystalline structures with various randomly oriented domains and boundaries. For semiconductors and dielectrics, boundary density can significantly affect the device's performance. To restrain the TMD domain boundaries, the epitaxial growth process plays a crucial role. It could become an ideal method if appropriate single‐crystal metal substrates like Au or Cu are selected.

Sapphire substrates have been broadly utilized to grow 2D monolayers due to their exclusive lattice constant and insulating surfaces.^[^ ^33^ ^]^ Numerous reports have embraced *c*‐plane sapphire as the substrate for developing 2D monolayers.^[^ ^34^ ^]^ However, it has been realized that the *c*‐plane sapphire surface was unsuccessful in terminating the generation of antiparallel domains and twin boundaries, resulting in the synthesis of polycrystalline films. Recently, the epitaxial growth of centimeter‐scale single‐crystal MoS_2_ monolayer on the Au (111) thin film has been reported.^[^ ^35^ ^]^ Despite this achievement, a significant drawback is the high cost of single‐crystal Au (111) and its limited size (≈1 cm) although it has the advantage of commercial availability. Further, it has been investigated that using single‐crystal Cu thin films is favorable to grow graphene film with no apparent grain boundaries and high carrier mobility.^[^ ^36^ ^]^ Meantime, the growth of a wafer‐scale single‐crystal hexagonal boron nitride (h‐BN) monolayer has been demonstrated on a Cu (111) thin film.^[^ ^37^ ^]^ Besides, it has also been noticed that graphene could also be developed along with a favorable orientation on Cu (111) with seamless stitching domains.^[^ ^38^, ^39^ ^]^ Therefore, the single‐crystal metal thin film becomes a favorable substrate for the growth of single‐crystal 2D monolayers.^[^ ^37^, ^39^, ^40^ ^]^ It will not be easy to grow a 2D TMD monolayer on Cu (111) thin film due to the catalytic nature of Cu. It tends to form CuS, which leads to the degradation of Cu. Therefore, only single‐crystal Au (111) thin film offers an excellent opportunity to grow 2D TMD monolayers with suppressed grain boundaries for various applications.^[^ ^41^, ^42^ ^]^ Eliminating the twin/grain boundaries of metal structures is also beneficial for electronic and optoelectronic devices. There are few works reported on the growth of 2D TMDs monolayer on a single crystal Au (111) thin films deposited on *c*‐plane sapphire by using the CVD technique.^[^ ^34^, ^43^ ^]^ Most recently, successful single‐crystal growth of wafer‐scale monolayer with excellent uniformity has been reported on vicinal *a*‐plane sapphire and miscut orientation toward *a*‐plane on *c*‐plane sapphire, inhibiting the nucleation for antiparallel TMD domains.^[^ ^44^, ^45^, ^46^, ^47^ ^]^ In this perspective, we first highlight the growth of single‐crystal metal using a single nucleation approach. Then, multi‐nucleation growth has been reviewed for growing large domain‐size single‐crystal 2D TMDs monolayers. Besides, we also outline the re‐utilization of the developed metal thin film after detaching successfully grown single‐crystal 2D TMD monolayers. In the end, progress and challenges are highlighted.

## 2 Growth Approaches

**Figure 1:**
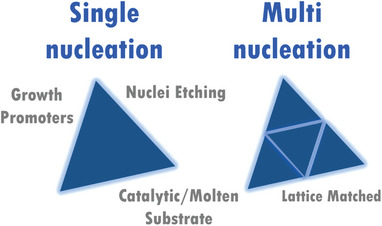
The approaches for growth of large domain‐sized single‐crystal 2D TMD monolayers.

Two major approaches, single‐nucleation and multi‐nucleation methods, have been reported for growing large domain‐size single‐crystal 2D monolayers (**Figure** **1**). In the single‐nucleation method, the nucleation density down to one nucleus has been achieved with the help of four techniques, including nuclei etching, utilizing substrates with a high catalytic capacity, choosing molten substrates as the growth assistant, and adding growth promotors (i.e., NaCl and KCl).^[^ ^48^, ^49^, ^50^, ^51^ ^]^ The best‐in‐class techniques have broadened the size of segregated single‐crystal TMD monolayers from 100 µm to >1 mm range. However, the significant drawback of such a vapor transport‐mediated growth method is that presenting only one single nucleus is extremely difficult. The subsequent domain size is still too small to be viable with the development of exceptionally integrated devices. On the other hand, the multi‐nucleation approach depends on a lattice‐matched substrate, which permits the epitaxial TMD domains to grow in a similar direction and later mix into a monocrystalline film.^[^ ^52^ ^]^ It allows the synthesis of numerous nucleation sites on the substrate, and a TMD single‐crystal film can thus be adaptable to the ideal size. **Table** **1** shows the electrical performance of 2D TMD FETs using various CVD methods, some of the representative works will be discussed in detail in the following sections.

**Table 1:** Electrical performance of 2D TMDs FET using various CVD methods.

Method	Material	Layer	Transfer characteristic	Carrier mobility [cm^2^ V^−1^ s^−1^]	On/off current ratio	Reference
Single nucleation (nuclei etching)	MoS_2_	Bilayer	p	29	10^4^	[55]
Single nucleation (nuclei etching)	MoS_2_	Monolayer	n	18.9	10^5^	[56]
Single nucleation (nuclei etching)	WSe_2_	Multilayer	p	500	10^4^	[57]
Single nucleation (catalytic substrates)	WS_2_/Au	Monolayer	n	20	10^8^	[58]
Single nucleation (catalytic substrates)	WSe	Monolayer	p	139	9.2 × 10^6^	[49]
Single nucleation (growth promotors)	MoS_2_	Bilayer	n	21	1.1 × 10^7^	[59]
Single nucleation (growth promotors)	WSe	Monolayer	n	26	10^7^	[60]
Single nucleation (molten substrates)	MoS_2_	Monolayer	n	8	10^7^	[61]
Single nucleation (molten substrates)	MoSe_2_	Monolayer	n	95	10^7^	[62]
Single nucleation (molten substrates)	WSe_2_	Monolayer	p	100	10^6^	[62]
Multi nucleation	MoS_2_	Monolayer	‐	82	2 × 10^10^	[13]
Multi nucleation	MoS_2_	Monolayer	n	90	10^7^	[48]

In this manner, the fundamental issue is how to initiate the uniform orientation of each domain. Examinations have been directed at developing enormous area TMD films on epitaxial substrates such as mica and sapphire.^[^ ^53^ ^]^ However, the sixfold symmetry of such substrates has been discovered to be incongruent with the threefold symmetry of TMD materials, typically prompting the development of antiparallel domains and unavoidable twin boundaries. Those boundaries are generally developed during the growth process and tend to work as conducting channels, which hinder the electrical and optical properties of related devices.^[^ ^54^ ^]^ Thus, controlling the domain orientation and decreasing the formation of twin boundaries are crucial in the growth of large‐scale TMD single‐crystal films. It has been studied that the underlying substrate plays an essential role in the growth of TMD films by suppressing twin boundaries. In this article, we will review both the growth approaches and their advantages and challenges.

## 3 Single‐Nucleation Methods

### 3.1 Nuclei Etching

**Figure 2:**
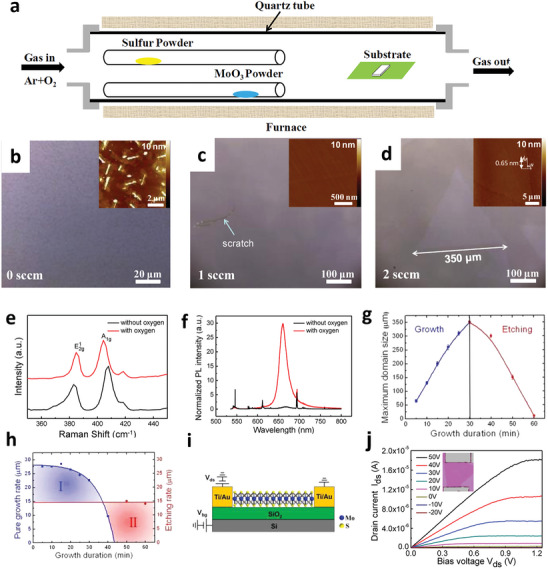
a) Schematic diagram of OA‐CVD. b–d) Optical and AFM images of MoS_2_ monolayers grown under various oxygen flow rates (0–2 sccm). e) Raman and f) photoluminescence (PL) spectra of MoS_2_ films grown with/without O_2_. g) Evolution of MoS_2_ domain size and variation of etching with the growth duration. h) Dependence of growth and the etching rates of domains as a function of the growth duration. i) Schematic of the fabricated monolayer MoS_2_ FET device on SiO_2_/Si substrate. j) Output characteristics of the FET device. Reproduced with permission.^[^ ^48^ ^]^ Copyright 2015 American Chemical Society.

Nuclei etching has drawn intensive attention to grow large domain‐size 2D TMD monolayers. Previously, Chen et al. have successfully reported the growth of large single‐crystal and high‐quality 2D MoS_2_ monolayer on the *c*‐plane sapphire substrate through nuclei etching using the oxygen‐assisted (OA) CVD technique.^[^ ^48^ ^]^ The schematic diagram of the OA‐CVD technique is shown in **Figure** **2**a. During the growth process, a mixture of Ar and a small amount of O_2_ was introduced as a carrier gas, and the pressure was kept at 0.5 Torr. It was observed that this small amount of O_2_ strongly reduced the MoS_2_ nucleation density, preventing poisoning of the MoO_3_ precursor for the large domain size growth of MoS_2_. Besides, it also led to eliminating defects that usually form during the growth process. In this work, the authors observed that the largest size of triangular shape single crystal domains were formed with side lengths ≈350 µm and very high room temperature mobility of 90 cm^2^ V‐s^−1^ on SiO_2_. The optical images of the MoS_2_ monolayers in Figure 2b–d reveal the growth of large triangular shape domains of 350 µm size at 2 sccm O_2_ flow rate (Figure 2c). In this work, authors have claimed that the observed domain size and mobility were higher than ever observed. The atomic force microscopy (AFM) results (insets of Figure 1b–d) of their work confirmed that a small amount of O_2_ could etch off unsteady nuclei and reduce the growth of nanoparticles/nanotubes.^[^ ^63^, ^64^ ^]^ The structural and optical properties of the MoS_2_ film before/after oxygen flow were observed by measuring Raman and photoluminescence (PL) spectra (Figure 2e,f), respectively, revealing the monolayer characteristics of MoS_2_.^[^ ^65^, ^66^ ^]^ They have also demonstrated the effect of oxygen flow during the growth process and investigated the growth of MoS_2_ with an O_2_ flow rate at 2 sccm with various growth durations. The average side lengths of grown domains as a function of growth durations are shown in Figure 2g.

It was observed that the maximum domain size and the nucleation density of the MoS_2_ film were gradually suppressed with further increase in the O_2_ flow rate. The relationship between the growth rates/etching of MoS_2_ domains as a function of growth durations was estimated and plotted in Figure 2h. The initial growth rate remains constant (<15 min) and drops gradually after the critical growth time. On the other hand, the etching rate was kept constant by maintaining the flow rate of O_2_. The MoS_2_ growth can be facilitated when the growth rate dominants over the etching rate. By further increasing the growth time (>30 min), the etching rate overcomes the growth rate, and the MoS_2_ domains shrink substantially. Hence, it was concluded that high quality MoS_2_ domains are highly associated with the synergistic effect of growth and etching.^[^ ^67^, ^68^ ^]^

Furthermore, the above studies have evaluated the electrical properties of MoS_2_ monolayers by fabricating FETs. The schematic of the fabricated device is shown in Figure 2i, and its output characteristics are presented in Figure 2j. The device exhibits n‐type behavior with linear *I‐V* characteristics (ohmic behavior) at low bias region. The estimated electron mobility of 90 cm^2^ V‐s^−1^ is twice the value of the reported mobility in the case of exfoliated and CVD‐grown MoS_2_ films. The high mobility can be mainly attributed to fewer sulfur vacancies, leading to less carrier trapping sites. Thus, nuclei etching using OA‐CVD shows much better quality on the growth of single‐crystal MoS_2_ monolayers. Despite these achievements, the disadvantage is that introducing only a single nucleus is extremely difficult, which limits the potential for large‐scale production.

### 3.2 Catalytic Substrates

**Figure 3:**
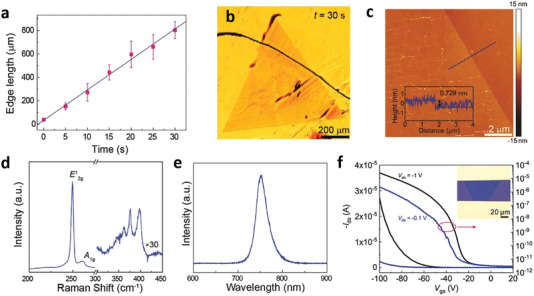
a) Growth‐time dependent edge length of single‐crystal monolayer WSe_2_ domains. b) Optical image of single‐crystal WSe_2_ domain on Au foil at t = 30 s. c) AFM image of the WSe_2_ domain, indicating a thickness of 0.73 nm. d) Raman and e) PL spectra of the WSe_2_ domain. f) Transfer characteristic of the WSe_2_ FET device. Reproduced with permission.^[^ ^49^ ^]^ Copyright 2017 Johns Wiley and Sons.

It has been noticed that catalytic substrates for promoting the rapid growth of 2D materials. Several metals have been widely used in CVD processes due to their high potential as catalysts. Recently, various catalytic metal substrates such as Au, Cu, and Cu‐Ni alloys have been explored for the ultrafast growth of single‐crystal graphene domains using CVD.^[^ ^69^, ^70^, ^71^ ^]^ For example, ultrafast growth of single‐crystal graphene on Cu substrate with a remarkable growth rate of 26 µm ^−1^s in the presence of oxygen has been reported to achieve graphene domains with lateral size ≈0.3 mm in 5 s.^[^ ^72^ ^]^ Besides catalytic metal substrates, 2D materials have also been grown on non‐metallic substrates such as mica, sapphire, and SiO_2_/Si based on the van der Waals epitaxial method.^[^ ^72^, ^73^, ^74^ ^]^ However, these substrates exhibit lattice mismatch and poor catalytic nature, resulting in various problems in terms of growth rate, domain orientation, uniformity, and controllability. Therefore, metal catalytic substrates such as Au and Cu have been admitted as ideal substrates for the ultrafast growth of high quality 2D materials.^[^ ^65^ ^]^ The ultrafast growth of high‐quality uniform tungsten diselenide (WSe_2_) monolayers on catalytic Au foil has been reported using ambient pressure CVD.^[^ ^49^ ^]^ The schematic of the CVD with a mounted precursor is shown in **Figure** **3**a. This work demonstrates the growth rate of ≈26 µm ^−1^s, which is 2–3 order of magnitude faster than those previously reported for the growth of 2D TMDs. Also, this growth rate is comparable to the highest growth rate of CVD‐grown graphene, which permits the growth of a large‐area single‐crystal WSe_2_ domain and a continuous monolayer film in 30 s (Figure 3b) and 65 s, respectively. The AFM image in Figure 3c reveals that the WSe_2_ domains exhibit a uniform thickness of ≈0.73 nm with an average surface roughness of ≈0.3 nm. The Raman spectra (Figure 3d) exhibit peaks ≈250 cm^−1^ (E^1^_2g_) and 261 (A_1g_) of WSe_2_ further confirm the monolayer characteristic and high thickness uniformity. The PL spectrum (Figure 3e) reveals a single fine peak at ≈752 nm with a half‐width at half maximum (FWHM) of ≈29 nm, which corresponds to the feature of a direct bandgap semiconductor.^[^ ^58^, ^61^, ^75^ ^]^

To evaluate the electrical properties of the ultrafast‐grown WSe_2_ domain, the as‐grown WSe_2_ was transferred from Au onto the SiO_2_/Si substrate using the electrochemical bubbling transfer method to fabricate back gate field‐effect transistors (FETs). The transfer characteristics (Figure 3f) represent the *p*‐type nature of the monolayer WSe_2_ semiconductor. The carrier mobility and corresponding ON/OFF ratio at applied V_ds_ = ‐1 V were ≈129 cm^2^ V‐s^−1^ and 9.2 × 10^6^, respectively. The carrier mobility is higher than the previously reported carrier mobilities (≈1–100 cm^2^ V‐s^−1^) of WSe_2_ monolayer directly grown on SiO_2_/Si substrates and close to the mobility (≈140–200 cm^2^ V‐s^−1^) observed in the mechanically exfoliated WSe_2_ film.^[^ ^76^, ^77^, ^78^, ^79^ ^]^ Despite the significant achievements, the drawback is a more significant trap density at the interface.^[^ ^80^ ^]^ Still, more efforts are required to suppress the interface trap density to enhance the performance of ultrafast‐grown WSe_2_ monolayer FETs devices.

### 3.3 Molten Substrates

Previously, the growth of the polycrystalline graphene film on a glass substrate was reported using the molten glass substrate method.^[^ ^81^ ^]^ The growth of graphene on the molten metallic substrate was also demonstrated to permit the formation of hexagonal crystallites to be gathered in an aligned orientation.^[^ ^62^ ^]^ Recently, a millimeter‐size high‐quality MoSe_2_ monolayer was demonstrated to be grown successfully on a molten glass substrate.^[^ ^50^ ^]^ In the study, it was suggested that the melting/regeneration of glass creates a smooth surface, thus enabling low‐density nucleation for significant crystal domain growth. The study claimed that triangular shape monolayer MoSe_2_ domains with a width in millimeters can be grown within 5 min. Using this approach, the growth of large‐area single‐crystal MoS_2_ monolayer was also achieved, indicating the universal suitability of molten glass substrate.

**Figure 4:**
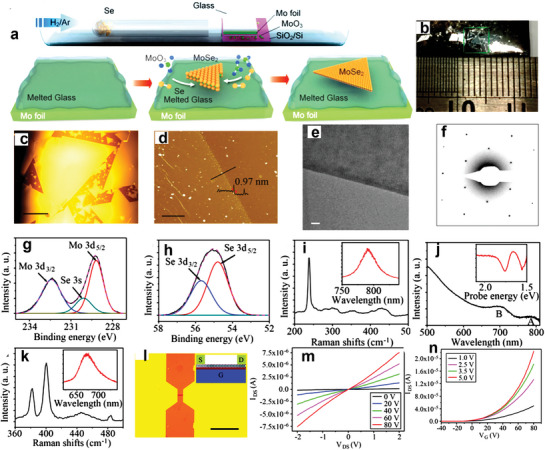
a) The growth mechanism of MoSe_2_ crystals on molten glass substrate using CVD. b) Photograph of MoSe_2_ crystals on a molten glass substrate. c) Optical image of MoSe_2_ crystals on a molten glass substrate (scale bar = 500 µm). d) AFM image of the MoSe_2_ crystal (scale bar = 1 µm). e) High‐resolution TEM image of the MoSe_2_ crystal (scale bar = 20 nm). f) SAED pattern of the MoSe_2_ crystal. g) XPS spectra of Mo 3d core levels. h) XPS spectra of Se 3d core levels. i) Raman and PL (inset) spectra of MoSe_2_ crystals. j) Absorption and transient absorption (inset) spectra of MoSe_2_ crystals. k) Raman and PL (inset) spectra of MoS_2_ crystals. l) Schematic of the fabricated MoSe_2_ FET. m) I_ds_‐V_ds_ curves of the FET. n) Transfer characteristics of the device as a function of V_ds_. Reproduced with permission.^[^ ^50^ ^]^ Copyright 2017 American Chemical Society.

The growth of high‐quality MoSe_2_ monolayer on molten glass was performed at room temperature using CVD, as shown in **Figure** **4**a. Glass substrates were inserted in a quartz tube placed inside the tube furnace to grow MoSe_2_. Glass with a Mo foil on the surface was first placed on a SiO_2_/Si with MoO_3_ precursor. During the heating process (up to 1050 °C), the solid glass was melted to form a clean and atomically flat surface. The melting of glass significantly suppressed the high‐energy interface trap sites, kinks, and asperities to further promote large‐area high‐quality MoSe_2_ crystal growth.^[^ ^62^ ^]^ Millimeter‐size MoSe_2_ triangular crystals on molten glass can easily be visually observed, as shown in Figure 4b,c. The results show large‐size domain formation with a uniform structure in the growth temperature range from 700 to 1050 °C. Upon increasing the temperature, the MoSe_2_ nucleation density reduced while domain size increased. At 1050 °C, the nucleation density is ≈20 nuclei cm^−2^. In addition, the growth can be further controlled by the H_2_ carrier gas. By increasing the H_2_ gas, the etching of MoSe_2_ crystals was facilitated; by reducing the H_2_ gas, the growth of hexagonally shaped MoSe_2_ crystals was enhanced. As‐grown MoSe_2_ crystals were transferred from the molten glass onto SiO_2_/Si substrate for AFM characterization (Figure 4d). The step height of ≈0.97 nm is consistent with the height of exfoliated MoSe_2_ monolayers.^[^ ^82^ ^]^

Furthermore, the authors investigated the microstructural properties of MoSe_2_ crystals using TEM (Figure 4e,f) and X‐ray photoelectron spectroscopy (XPS) (Figure 4g,h). The SAED of MoSe_2_ crystals reveal identical orientation of diffraction patterns for various spots on the crystal, confirming the single crystal nature of MoSe_2_. The results were also confirmed with low‐energy electron diffraction (LEED) and scanning TEM. The elemental compositions and bonding among Mo and Se were investigated by recording the XPS spectra of MoSe_2_. The XPS spectra contain the binding energy peaks of Mo 3d_3/2_ (231.5 eV), Mo 3d_5/2_ (228.3 eV), Se 3d_5/2_ (55 eV), and Se 3d_3/2_ (55.9 eV) core levels. The MoO_3_ peaks were not observed, indicating low defect density at the surface. The integrated peaks area reveals the desired stoichiometry (1:2) of Mo and Se in MoSe_2_ crystals. The crystal quality and optical properties of MoSe_2_ crystals were further investigated by Raman, PL, and absorption spectra shown in Figure 4i,j.^[^ ^83^, ^84^ ^]^ Both Raman and PL results show evidence of single crystal characteristics of as‐grown MoSe_2_ on molten glass with high thickness uniformity.

To evaluate the potential of the method in other 2D TMDs, it was also applied to grow MoS_2_ crystals. The approach and parameters were similar to MoSe_2_ growth, aside from the fact that S (instead of Se) was utilized as a source. The Raman and PL spectra of the as‐grown MoS_2_ crystal are shown in Figure 4k. The Raman bands observed ≈400 cm^−1^ and 382 cm^−1^ correspond to the A_1g_ and E_2g_ modes of MoS_2,_ respectively. The PL characteristic emission peak at ≈673 nm indicates the direct bandgap excitonic transition of MoS_2_ monolayer. In this work, the size of the as‐grown MoS_2_ domain can reach up to 2.6 mm, which is relatively higher than domain size of 2D TMDs grown using different growth approaches.^[^ ^63^, ^73^ ^]^

The electrical performance of the MoSe_2_ crystal was investigated by fabricating FETs on the SiO_2_/Si substrate (Figure 4l). The *I‐V* characteristic of the FET with a channel length (L) of ≈10 µm and width (W) of 2 µm is shown in Figure 4m. Figure 4n shows the transfer characteristics of the device as a function of V_ds_, revealing a gradual increase in the I_ds_ with increasing the V_gs_ bias voltage (*n*‐type behavior). Further, the ON/OFF ratio and threshold voltage were found to be ≈10^7^ and ≈‐20 V (Figure 4n), respectively. The carrier mobilities by and ON/OFF ratios from 19 devices have also been extrated and found to be in the range of 5–95 cm^2^ V‐s^−1^ and ≈10^4^–10^7^, respectively. These values are comparable to the previously reported ones in the case of mechanically cleaved MoSe_2_, suggesting the high crystal quality of MoSe_2_ crystals grown by the molten glass approach.^[^ ^85^, ^86^ ^]^

### 3.4 Growth Promotors

One of the major challenges in the production of large‐scale and high‐quality 2D TMDs is the high melting point of metal and metal oxide precursors. Therefore, molten‐salt‐assisted methods have been employed over the past several years to promote the growth of 2D TMD monolayers at relatively low temperatures.^[^ ^60^, ^87^ ^]^ It has been discovered that the molten‐salt‐assisted CVD can be widely utilized to synthesize ≈47 compounds including 32 binary compounds, 13 alloys, and a couple of 2D heterostructures.^[^ ^51^ ^]^ In the study, salt has been demonstrated to reduce the melting point of the reactants, promote the development of intermediate products, and enhance the overall reaction rate.

The mass flux decides the quantity of metal precursors associated with the nucleation process, while the growth rate affects the grain size of the as‐grown film. High mass flux and low growth rate lead to polycrystalline films formed with small grain size. However, high mass flux and high growth rate lead to the formation of a continuous film with large grains (≈millimeters).^[^ ^88^ ^]^ Conversely, low mass flux and low growth rate tend to form small flakes. It was suggested that tiny nuclei were frequently found in the middle of flakes, which require extra adatoms or atom clusters to be reliably bounded to an existing nucleus during the growth process.^[^ ^89^ ^]^ Further, low mass flux and high growth rate assist in forming an individual large‐area single‐crystal 2D TMD domain.^[^ ^90^ ^]^ It was also observed that some TMDs, such as those made of Nb, Pt, and Ni, were difficult to synthesize. The high melting points and low vapor pressures of these metal or metal oxide precursors result in low mass flux to limit the reaction process. Therefore, molten salt was used to increase the mass flux by producing oxychlorides through the reaction with certain metal oxides, reducing the melting point of metal precursors, and enhancing the reaction rate. The growth mechanism of the molten‐salt‐assisted CVD was demonstrated using salt (as a growth promotor) that could decrease the melting point of metal precursors and therefore make the reaction possible. For instance, a correlation of Nb nucleus with and without salt was added, demonstrates a high mass flux of metal precursors facilitated by the salt, notwithstanding the downfall in the melting point. The existence of metal oxychloride has also been investigated by analyzing the intermediate products through XPS and density function theory (DFT) calculations. It was found that the sulfurization of metal oxychlorides is extremely energetically preferable than the sulfurization of metal oxides. Therefore, salt‐assisted CVD can be used as a universal approach for the growth of various 2D TMDs, which holds much potential for industrial applicability.

## 4 Multi‐Nucleation Methods

**Figure 5:**
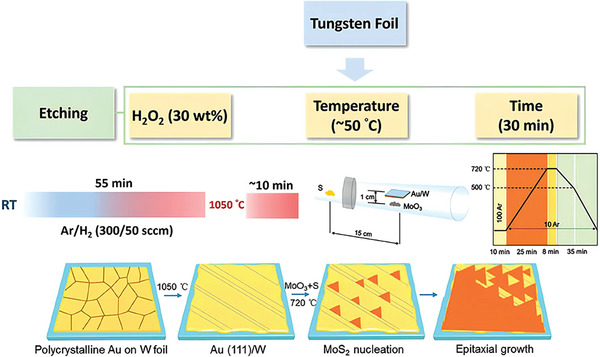
The growth process of single‐crystal metal Au (111) on W, MoS_2_ monolayer on Au (111)/W, the schematic of the CVD quartz tube, and the schematic illustration of continuous MoS_2_ film growth during CVD. Reproduced with permission.^[^ ^43^ ^]^ Copyright 2020 American Chemical Society.

To obtain the controllable growth of wafer‐scale single crystal 2D TMDs with high crystallinity, the lattice‐matched approach was demonstrated to become a promising method for large‐scale production.^[^ ^43^, ^88^ ^]^ Recently, epitaxial growth of 100 cm^2^ single‐crystal hexagonal boron nitride (h‐BN) monolayer has been reported on a single‐crystal vicinal Cu (100) surface.^[^ ^35^ ^]^ It was observed that a unidirectional alignment of the h‐BN appeared through the coupling of h‐BN zigzag edge with the Cu (211) step edge. Subsequently, epitaxial growth of single‐crystal h‐BN monolayer was introduced on Cu (111) thin film at a temperature ≈1050 °C using the ammonia borane precursor in the presence of H_2_ gas. The step edge of single‐crystal Cu (111) was introduced to induce the mono‐orientation growth of h‐BN monolayers. It was also theoretically realized that the equivalence 0 to 60° domains can be broken if the surface of the substrate is vicinal.^[^ ^91^ ^]^ A similar study has been reported on the growth of identically oriented WS_2_ domains on a single crystalline h‐BN layer on a melted gold surface.^[^ ^92^ ^]^ This work provided the possibility of growing large‐area single‐crystal 2D TMD monolayer on single crystal metallic substrates. Quite recently, single‐crystal 2D MoS_2_ monolayers have been successfully grown on Au (111) thin film using CVD.^[^ ^43^ ^]^ Before the CVD process, a vicinal Au (111) thin film was first grown on a tungsten (W) substrate through the melting and re‐solidifying process of commercially available Au foils (**Figure** **5**). However, the re‐solidified Au on the W substrate formed a polycrystalline film. After the post thermal annealing process, single‐crystal Au (111) film can be obtained. Generally, Au (111) forms twin grains isolated through twin boundaries during growth kinetic process. Therefore, optimized temperature (1040–1080 °C) was proposed in the presence of Ar/H_2_ (300/50 sccm) gas, revealing key factors for eliminating the twin grains.

**Figure 6:**
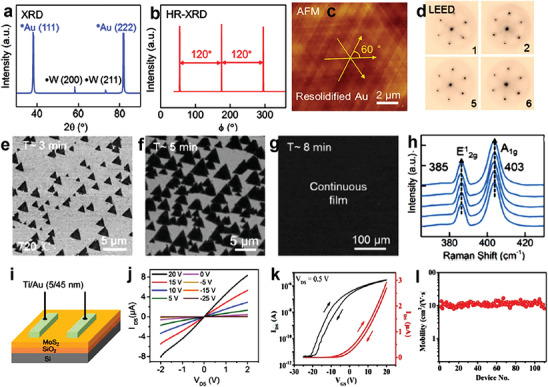
a) XRD pattern of the Au/W foil. b) HR‐XRD azimuthal *ϕ* scans of the off‐axis ⟨111⟩ reflection of the Au (111) film. c) AFM image of the re‐solidified single‐crystal Au (111) substrate. d) LEED pattern of the MoS_2_ continuous film collected at various positions. e) SEM image of identically oriented small MoS_2_ domains at the growth time of 3 min. f) SEM image of identically oriented extended MoS_2_ domains at the growth time of 5 min. g) SEM image of the continuous MoS_2_ film at the growth time of 8 min. h) Raman spectra of MoS_2_ film on Au (111)/W film at various positions. i) Schematic of the single‐crystal MoS_2_ FET device. j) I_DS_‐V_DS_ characteristics of the single‐crystal MoS_2_ FET device. k) I_DS_‐V_GS_ curves of the single‐crystal MoS_2_ FET device. l) The distribution of carrier mobilities over 110 FET devices. Reproduced with permission.^[^ ^43^ ^]^ Copyright 2020 American Chemical Society.

To grow Au (111) film on W (etched in 30 wt% H_2_O_2_), a piece of gold foil was first mounted on the etched W foil. This structure was heated up to 1050 °C from room temperature within 55 mins and maintained for 10 min at 1050 °C in the presence of Ar/H_2_ (300/50 sccm) mixed gas. Eventually, the molten gold liquid spreads on the surface of the W foil. After cooled down, the liquid gold solidified and formed steps on the Au (111) surface. Later, single‐crystal MoS_2_ was grown on the face‐down Au (111)/W substrate using the CVD method. To confirm the formation of the Au (111) plane, X‐ray diffraction (XRD) and high resolution (HR)‐XRD patterns of the Au/W film have been obtained and shown in **Figure** **6**a,b, respectively. The XRD pattern of the Au/W film comprises four diffraction peaks which associate with (111) and (222) planes of Au and (200) and (211) planes of W. This demonstrates the growth of a single‐crystal Au (111) film on the polycrystalline W foil. Also, the HR‐XRD pattern reveals only three peaks with an interval of 120° in the *ϕ* scan. In the case of face‐centered cubic (FCC) metals, (111) exhibits minimum surface energy among all the planes.^[^ ^93^ ^]^ The melting of gold into a liquid state could generally reduce the thermal pressure from its interfacial contact with bare W foils. During the cooling process, Au (111) tends to form for achieving surface energy reduction. Further, the formation of single‐crystal Au (111) film has also been confirmed by measuring its topography using AFM, as shown in Figure 6c. The AFM image shows the atomic step is aligned in three directions, including a crossing angle of 60°.

To demonstrate the single‐crystal nature of MoS_2_ grown on the Au (111)/W substrate, low‐energy electron diffraction (LEED) patterns were recorded at various positions (Figure 6d). The results reveal identical lattice orientations, indicating the single‐crystal nature of the MoS_2_ film. The SEM (Figure 6e) image shows the formation of triangular and well‐oriented aligned domains on the Au (111) substrate (synthesized at ≈720 °C for 30 min). It was also found that the proportion of aligned domains was as high as ≈98%. The unidirectional domains merged (Figure 6f) upon increasing the growth time and eventually converted into a continuous MoS_2_ film (Figure 6g). The Raman measurement (Figure 6h) was performed at five different positions on MoS_2_ and revealed similar peak positions ≈385 and 403 cm^−1^, indicating monolayer characteristics and high thickness uniformity. To understand the electrical properties of the as‐grown single‐crystal MoS_2_ film on Au (111)/W, the MoS_2_ film was transferred to the SiO_2_/Si substrate to fabricate a FET (Figure 6i). It was noted that the Au/W template is chemically inert and may be re‐utilized after the transfer process. The current‐voltage characteristics of FETs (channel width and length of 15 and 5 µm) were measured and shown in Figure 6j, which reveals the *n*‐type behavior. Figure 6k shows the I_DS_‐V_GS_ curve at V_DS_ = 0.5 V. The ON‐OFF ratio exceeds 10^5^ has been observed. Figure 6l shows the carrier mobility (≈11.2 cm^2^ V‐s^−1^) over 110 devices. Although the mobility is not extremely large compared with previous results on single nucleation 2D TMD domains, the lattice‐matched multi‐nucleation method shows the potential for wafer scale, substrate reusable, and reliable growth technique that would be more preferred in industrial applications.

## 5 Conclusion and Outlook

This perspective makes a significant step toward understanding main approaches on single and multiple nucleations for large‐scale 2D TMD preparation. Investigating the underlying phenomenon of the uniform growth of 2D TMD monolayers on single‐crystal film is still a fundamental issue in related fields. In this perspective, we have first reviewed the approach of single nucleation growth through which the nucleation density down to a single nucleus is possible with the help of four major methods: (i) nuclei etching, (ii) utilizing substrates with high catalytic capacity, (iii) choosing molten substrates as growth assistant, and (iv) growth promotors. Despite the major success being achieved in single nucleation for designing millimeter‐size high quality single crystal FET devices with high mobility and large ON/OFF ratio, it seems to be extremely difficult to go beyond this scale. Thus, the multi‐nucleation method should be the mainstream for developing the industrially applicable route for achieving wafer‐scale 2D film production in the next several years. However, multi‐nucleation strategies in the past few years require single‐crystal metallic substrate such as Au(111) prior to the growth procedure, which could largely increase the cost of the production process. In addition, the fabrication of devices such as FETs requires the 2D film to be further transferred to other insulating substrates, which may possibly affect the uniformity, integrity, and internal strain of the film. Nonetheless, the strategy indeed opens a promising new path for the possibility of large‐scale high‐quality 2D film production. The recently proposed wafer‐scale epitaxial growth of monolayer TMD on vicinal *a*‐plane or miscut *c*‐plane sapphires indeed opens a new avenue in developing appropriate substrates to efficiently inhibit antiparallel growth as well as twin boundaries to achieve large‐scale uniformity. By tuning the miscut angle, vicinal step density can be further controlled. Although the development of such strategy is still under progress, we believe that similar design will dominate the growth of wafer‐scale single crystal monolayer TMD by evaluating various insulating substrate materials, miscut (off‐cut) angles, growth/substrate pre‐annealing temperatures, and TMD materials. It can be expected that in the near future, similar techniques combining the lattice‐matched dielectric substrate, effective CVD (plasma‐enhanced CVD or metal‐organic CVD), clean and strain‐free transfer methods, and appropriate catalysts will be on the way for the development of commerically available high‐quality single‐crystal TMD films.

## Conflict of Interest

The authors declare no conflict of interest.
